# Intravascular forward-looking ultrasound transducers for microbubble-mediated sonothrombolysis

**DOI:** 10.1038/s41598-017-03492-4

**Published:** 2017-06-14

**Authors:** Jinwook Kim, Brooks D. Lindsey, Wei-Yi Chang, Xuming Dai, Joseph M. Stavas, Paul A. Dayton, Xiaoning Jiang

**Affiliations:** 1 Department of Mechanical and Aerospace Engineering, North Carolina State University, Raleigh, NC 27695 USA; 2 Joint Department of Biomedical Engineering, University of North Carolina and North Carolina State University, Chapel Hill, NC 27599 USA; 3 Division of Cardiology, University of North Carolina, Chapel Hill, NC 27599 United States; 4 Division of Vascular and Interventional Radiology, University of North Carolina, Chapel Hill, NC 27599 United States; 5 Department of Mechanical and Biomedical Engineering, City University of Hong Kong, Hong Kong, China

## Abstract

Effective removal or dissolution of large blood clots remains a challenge in clinical treatment of acute thrombo-occlusive diseases. Here we report the development of an intravascular microbubble-mediated sonothrombolysis device for improving thrombolytic rate and thus minimizing the required dose of thrombolytic drugs. We hypothesize that a sub-megahertz, forward-looking ultrasound transducer with an integrated microbubble injection tube is more advantageous for efficient thrombolysis by enhancing cavitation-induced microstreaming than the conventional high-frequency, side-looking, catheter-mounted transducers. We developed custom miniaturized transducers and demonstrated that these transducers are able to generate sufficient pressure to induce cavitation of lipid-shelled microbubble contrast agents. Our technology demonstrates a thrombolysis rate of 0.7 ± 0.15 percent mass loss/min *in vitro* without any use of thrombolytic drugs.

## Introduction

Deep vein thrombosis (DVT) is the formation of blood clots within the deep veins of the legs, either in the calf or more proximally in the popliteal, femoral, or iliac veins^1^. The most serious complication of DVT is pulmonary embolism (PE), which can occur when a blood clot detaches from vein walls, travels through the heart to the lungs, and occludes pulmonary arteries^2^. There are more than 0.1 million cases of PE annually in the U.S. alone^3^, with 20–25% of cases resulting in sudden death^4^. Additionally, PE often causes considerable morbidity and health care costs for hospitals and survivors^5–7^. In high-risk cases of PE (i.e. where there is persistent hypotension or shock and evidence of right ventricular dilation and dysfunction), pharmacological dissolution using recombinant tissue plasminogen activator (rt-PA), catheter-directed mechanical fragmentation or surgical removal may be utilized^8, 9^. These current techniques for treating severe PE are plagued by limitations such as low thrombolytic efficiency, frequent bleeding complications, high failure rate, vein injury-associated severe regional dysfunction, high recurrence rates, and the risk of distal embolism due to the relatively large size of clot debris^10–13^. In order to overcome such limitations, ultrasound-enhanced thrombolysis, also called sonothrombolysis, has been used as an alternative therapy promoting efficient thrombus dissolution without increasing the risk of systemic bleeding complications^14^. Specifically, application of ultrasound has shown the potential for enhancing both clot permeability to rt-PA and mechanical damage to the clot by cavitation-induced microstreaming without the use of thrombolytic agents^15, 16^.

Among various ultrasound-delivery methods for sonothrombolysis, catheter-delivered transducer tipped ultrasound has exhibited several advantages including efficient delivery of acoustic energy, flexible frequency control, and negligible ultrasound-induced heating on surrounding tissue^14^. Recently, catheter-based side-looking intravascular ultrasound thrombolysis (e.g. Ekosonic, EKOS Corporation, Bothell, WA, USA) have improved lytic efficiency by using pulsed high-frequency (2 MHz), low-power ultrasound waves^17, 18^. In this catheter system, side-looking transducer arrays mounted in a catheter penetrate a target clot, producing a circumferential insonation region. While the ultrasound itself cannot dissolve the target clot due to its low acoustic power, penetration of rt-PA into the clot is accelerated by the applied ultrasound energy^18^. However, this technique still suffers from long treatment times (16 hrs in average), and the lytic rate is highly dependent on the total dose of thrombolytic agent delivered, which is limited due to concern over the harmful hemorrhagic effects of rt-PA^19^. Moreover, some randomized controlled clinical trials have shown no difference in thrombolytic efficacy for Ekosonic catheters compared to conventional catheter directed thrombolysis (CDT)^20–22^, possibly because pulsed low-power ultrasound (<0.5 W/cm^2^) might be insufficient to realize ultrasound-enhanced thrombolytic efficiency^20, 23^. Although it has been comprehensively demonstrated that general sonothrombolysis with higher power, lower frequency ultrasound yields higher thrombolytic rate^24^, the design of the side-looking intravascular transducer limits its ability for use with optimal sonothrombolytic acoustic parameters due to the orientation of the transducer and resulting propagation direction toward the vessel wall. Direction of the high intensity ultrasound energy directly towards the vessel wall increases the likelihood of healthy tissue damage from overexposure to acoustic energy. Moreover, a larger-diameter catheter is required for the lower-frequency transducer because the frequency-dependent dimension is parallel to the catheter diameter. We hypothesize that a forward-looking design will enable generation of higher pressures at a lower operating frequency, which can in turn enhance the lytic rate and reduce the amount of rt-PA required. This design will also limit the likelihood of catheter-clot contact while directing acoustic energy towards the clot rather than directly towards the vessel wall, thus reducing the risk of injury to the vessel wall and minimizing the likelihood of accidental dislodging of large clot particles which could cause emboli.

Herein, we describe the development of customized intravascular forward-looking ultrasound transducers for low-frequency (<1 MHz), moderate-power ultrasound to improve the thrombolysis rate and to minimize the required dose of rt-PA. Moreover, we adopted a microbubble contrast agent (MCA) –mediated sonothrombolysis approach for enhanced cavitation. As microbubbles distributed within the ultrasound beam and in close proximity to the target clot act as nuclei for cavitation, the pressure threshold is reduced, thereby resulting in improved lytic rate with a lower ultrasound exposure^25, 26^. In this proof-of-concept study, we aim to demonstrate the feasibility of intravascular forward-looking ultrasound transducers for microbubble-mediated sonothrombolysis *in vitro*.

## Results

### Intravascular sonothrombolysis device and system

**Figure 1:**
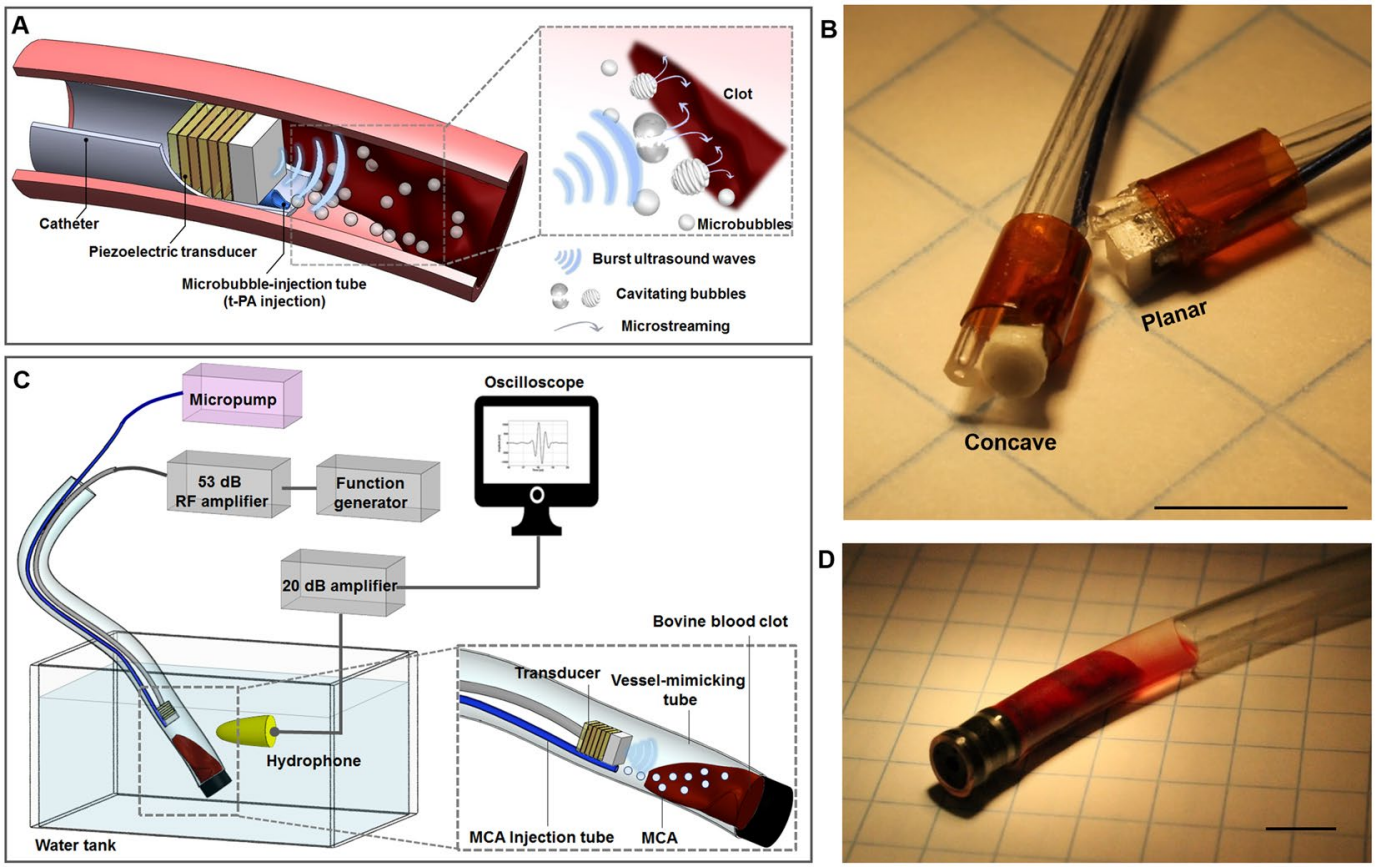
Microbubble-mediated intravascular sonothrombolysis system. (**A**) Principle of microbubble-mediated intravascular sonothrombolysis by using a catheter-tipped forward-looking transducer; low-frequency ultrasound waves generate stable and inertial cavitation of locally injected microbubbles near the target blood clot. (**B**) Customized 620 kHz stacked piezoelectric transducers integrated with a microbubble-injection tube and polyimide housing: concave- and planar-aperture (lateral dimension <1.5 mm) prototypes (scale bar = 5 mm). (**C**) *In vitro* experiment setup; a tygon tube (ID: 4 mm, OD: 5.6 mm) was used as a vessel-mimicking structure. The hydrophone was used to detect the microbubble response. (**D**) Bovine blood clot sample (200 mg ± 10%) located in the tygon tube with 50 μl saline water (scale bar = 5 mm). The figures (**A**) and (**C**) were drawn through the combined use of Solidworks Education Edition, Microsoft PowerPoint, and Adobe Illustrator CC. The figures (**B**) and (**D**) were captured using a digital SLR camera (Canon EOS Rebel T3) with 50 mm lens (Canon, f/2.5 Compact Macro Lens), cropped and aligned using Microsoft PowerPoint and Adobe Illustrator CC.

The catheter size for PE treatments is about 6 F to 11 F (i.e. 2 to 3.7 mm in diameter)^27, 28^, so the custom catheters described herein composed of a miniaturized forward-looking transducer (lateral dimension <1.5 mm) and a microbubble injection tube (outer diameter of 1.1 mm), were developed as an 8F-prototype catheter (diameter of 2.7 mm). With this catheter device, sub-megahertz frequency ultrasound waves are excited to yield stable and inertial cavitation of locally-injected microbubbles near a target blood clot (Fig. 1(A)). The main thrombolysis mechanism is cavitation-induced microstreaming, which causes shear stress on the clot structure^29, 30^. Note that the present system excludes rt-PA injection, although it is likely that therapy using this system would involve low-dose use of thrombolytic drugs to avoid distal embolism caused by the fragmented clot particles. We anticipated that discernible improvement in sonothrombolysis efficiency without any thrombolytic agents can be a useful indicator of ultrasound-enhanced fibrinolysis by using our device since the accelerated efficiency arising from improved rt-PA penetration in MCA-involved therapy has been previously reported in detail^16, 31^. In order to attain sufficient pressure output for the bubble cavitation at sub-megahertz frequency, stacked-type piezoelectric transducers were designed and fabricated with the resonance frequency of 620 kHz (Fig. 1(B)). Although the low operating frequency is advantageous in yielding inertial cavitation of the MCA, the wide beam width due to the low frequency is inappropriate for precise insonation. Hence, we built a custom concave lens made of an aluminum oxide (Al_2_O_3_)/epoxy mixture for beam focusing to the target clot, minimizing the exposure area of the vessel wall. More detailed transducer design and fabrication procedures are provided in the Supplementary material (S1 and S2). The fabricated transducers with and without the concave lens were assembled with a microbubble-injection tube and 10 AWG-polyimide housing (outer diameter of 2.7 mm). The developed sonothrombolysis system is shown in Fig. 1(C). Thrombolysis efficiency of the developed system was evaluated *in vitro*. A bovine blood clot sample (200 mg ± 10%) was stored in a vessel mimicking tube (Fig. 1(D)), and the transducer was positioned close to the target clot (<0.5 mm from the aperture) using a 3-axis motion stage.

### Characterization of the prototype transducers

**Figure 2:**
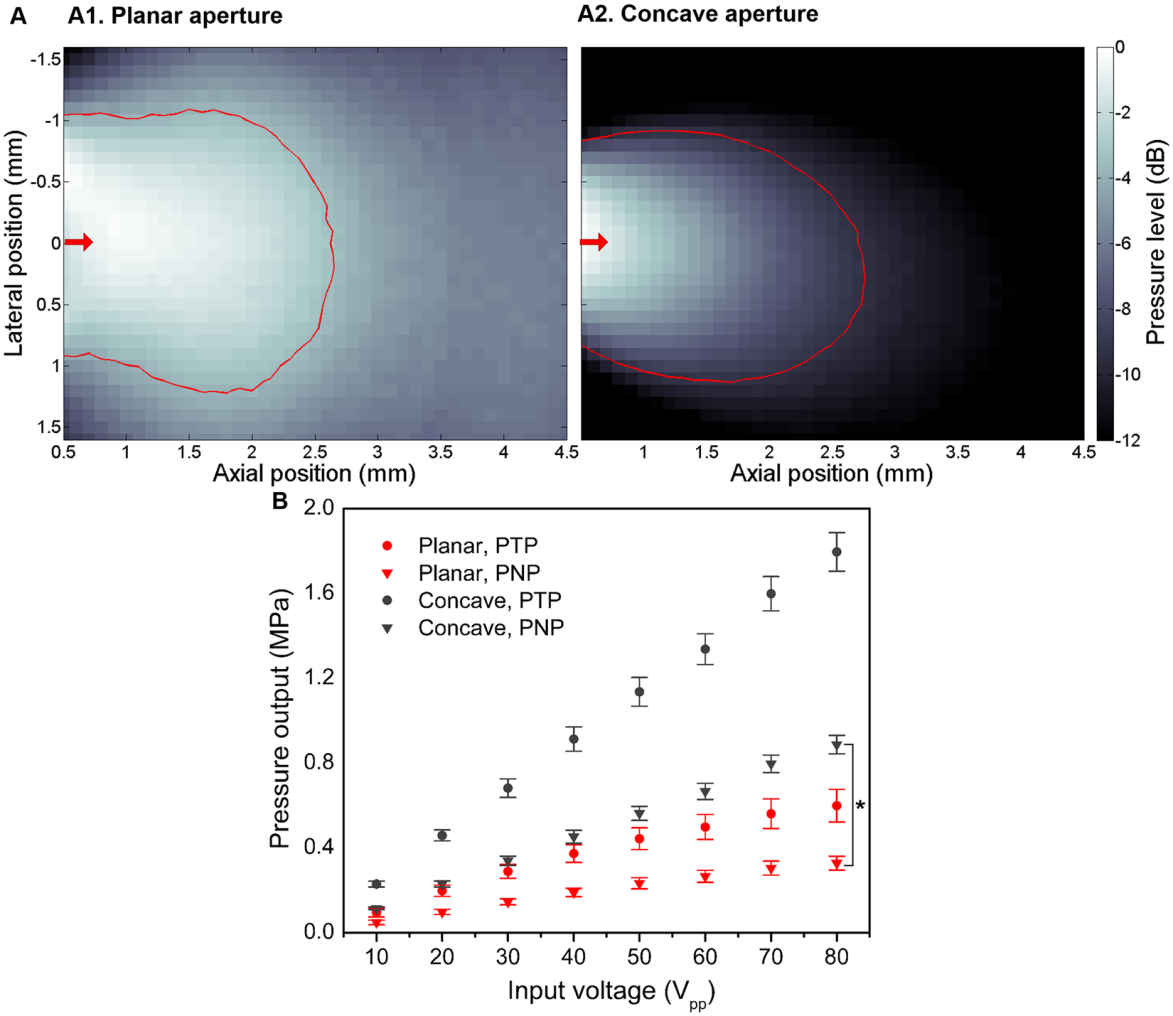
Acoustic characterization of customized stacked-type intravascular ultrasound transducer. (**A**) Measured beam profiles of planar- (A1) and concave-aperture (A2) prototypes; the red arrows indicate the radiation direction, and the red contour lines denote the mechanical index of 0.3 with voltage input of 80 V_pp_ which implies the approximated threshold of inertial cavitation of microbubbles. −6 dB beam diameters of planar- and concave-aperture prototypes are 3.1 mm and 1.3 mm, respectively. (**B**) Measured pressure output (1 mm away from the aperture) with voltage inputs of 10–80 V_pp_ (n = 2). The concave aperture prototype exhibits approximately 2.5-fold increase in peak-to-peak pressure (PTP) and 2.3-fold increased peak-negative-pressure (PNP) relative to the planar aperture prototype. (*P < 0.05).

The prototype transducers were acoustically characterized to confirm that the acoustic pressure is sufficient for cavitation of the injected microbubbles in the confined field of insonation. Beam profiles of both planar and concave aperture-prototypes are shown in Fig. 2(A). −6 dB beam diameters of planar- and concave-aperture prototypes are 3.1 mm and 1.3 mm, respectively. We confirmed that the concave lens is able to confine the beam width despite the small aperture area in terms of wavelength. Although theoretical design guidelines for the focusing lens recommend large aperture diameters (>5λ), in practicality, lenses can be designed with rather modest aperture/wavelength ratios^32^. Due to the small aperture, the measured beam, which was designed to have a 1 mm focus based on the geometry of concave lens, exhibited a diffuse focal region rather than a single focal distance. However, the focused aperture showed a spatially confined beam compared to the planar transducer. In order to evaluate the transmitting sensitivity, the measured pressure output (1 mm away from the aperture) of both planar and concave transducers were compared for various voltage inputs as shown in Fig. 2(B). Voltage inputs were varied in the range of 10–80 V_pp_. The concave aperture prototype exhibited approximately 2.5-fold higher peak-to-peak pressure (PTP) and 2.3-fold higher peak-negative-pressure (PNP) outputs than the planar aperture prototype. One quantitative metric related to microbubble-mediated sonothrombolysis is mechanical index (MI), which is defined as PNP (in MPa) divided by the square root of the operating frequency (in MHz). Within the voltage input range, the maximum MI with planar and concave aperture design were 0.4 and 1.0, respectively. For clinical imaging, microbubble contrast agents are approved for use in humans at MIs up to 0.8 (Definity, Lantheus Medical Imaging, North Billerica, Massachusetts, formerly Bristol-Myers Squibb Medical Imaging), though they are often flashed with short bursts at higher MI in destruction-reperfusion imaging studies^33–35^. Mechanical indices which exceed this imaging limit have been reported in sonothrombolysis^36–38^. It was previously reported that the approximate MI threshold for MCA cavitation is 0.35^39^ when the excitation frequency is lower than microbubble resonance frequency (1–5 MHz for MCA with an approximated microbubble diameter of 1 μm), and we confirmed in this work that both prototypes with different aperture designs are able to yield inertial cavitation during microbubble-mediated thrombolysis therapies.

### *In vitro* tests

**Figure 3:**
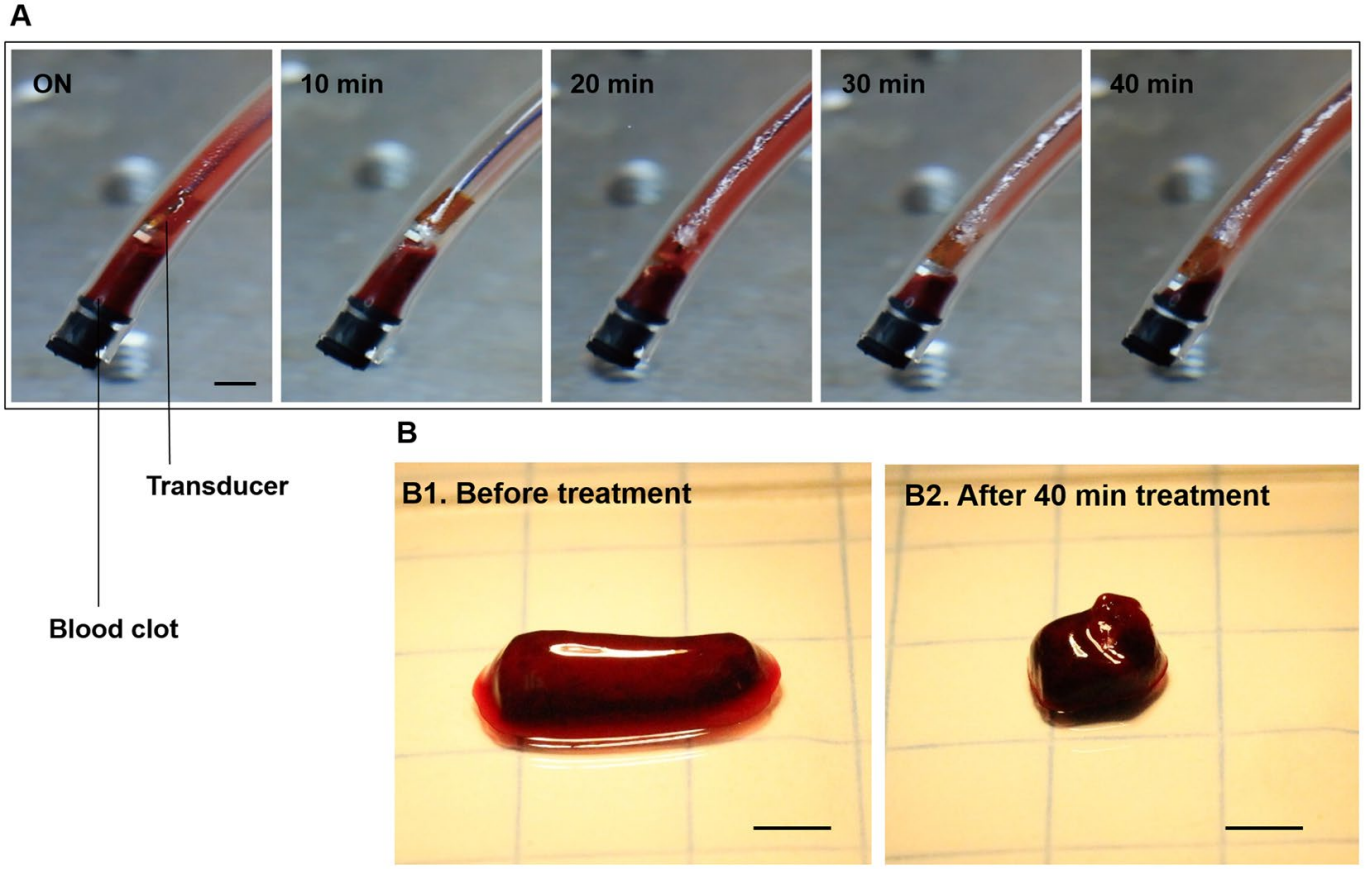
*In vitro* clot lysis by microbubble-mediated intravascular ultrasound. (**A**) Captured images during 40 min treatment by using the planar-aperture prototype (input conditions of 620 kHz, 80 V_pp_, 10% duty cycle with 100 μl/min injection of 50^8^ bubbles/ml). A smaller (120 mg) clot was used for monitoring the volume reduction (scale bar = 3 mm). Due to the high bubble concentration (50^8^ bubbles/ml) we observed that the undestroyed bubbles were attached to the transducer and the inner surface of the tygon tube. (**B**) A 120 mg clot was reduced to 50 mg after the 40 min treatment (scale bar = 3 mm).

We investigated the reduction in volume of the bovine blood clot using the developed sonothrombolysis system with the planar lens prototype transducer. Initial *in vitro* tests were conducted using a 120 mg clot positioned in a tygon tube (Fig. 3(A)). The captured images with 10 min-interval show gradual volume reduction. The 40 min treatment includes a 30 sec break for every 5 min of sonication in order to allow new microbubbles penetrate into the fibrin clot^40^. After a 40 min treatment, the target clot size reduced to about 35% of its original size (mass reduction from 120 mg to 50 mg, Fig. 3(B)). The position of the transducer was controlled to keep the face of the transducer less than 0.5 mm from the clot surface. Since relatively high concentrations of MCA (50^8^ bubbles/ml) were used in this initial test, we observed some groups of undestroyed microbubbles attached to the transducer and the inner surface of the tygon tube during the treatment even though the output pressure with the 80 V_pp_ input (PNP of 250 kPa) is sufficient to destroy the bubbles. This remaining population may include microbubbles which have coalesced into larger microbubbles^41^. Observed remaining bubbles may also be due to the smaller cavitation zone compared to the inner diameter of the vessel-mimicking tube and the period during which bubbles were injected without insonation. Despite the very low (100 μl/min) bubble infusion rate, intact bubbles can be spread around the vessel wall during 30 sec infusion without insonation. During treatment with a 10% duty cycle, the temperature at the clot boundary (insonation area) remained unchanged as measured by calibrated thermocouple (37 °C). This result indicates that the clot was dissolved by cavitation-induced microstreaming without measurable ultrasound-induced thermal effects.

**Figure 4:**
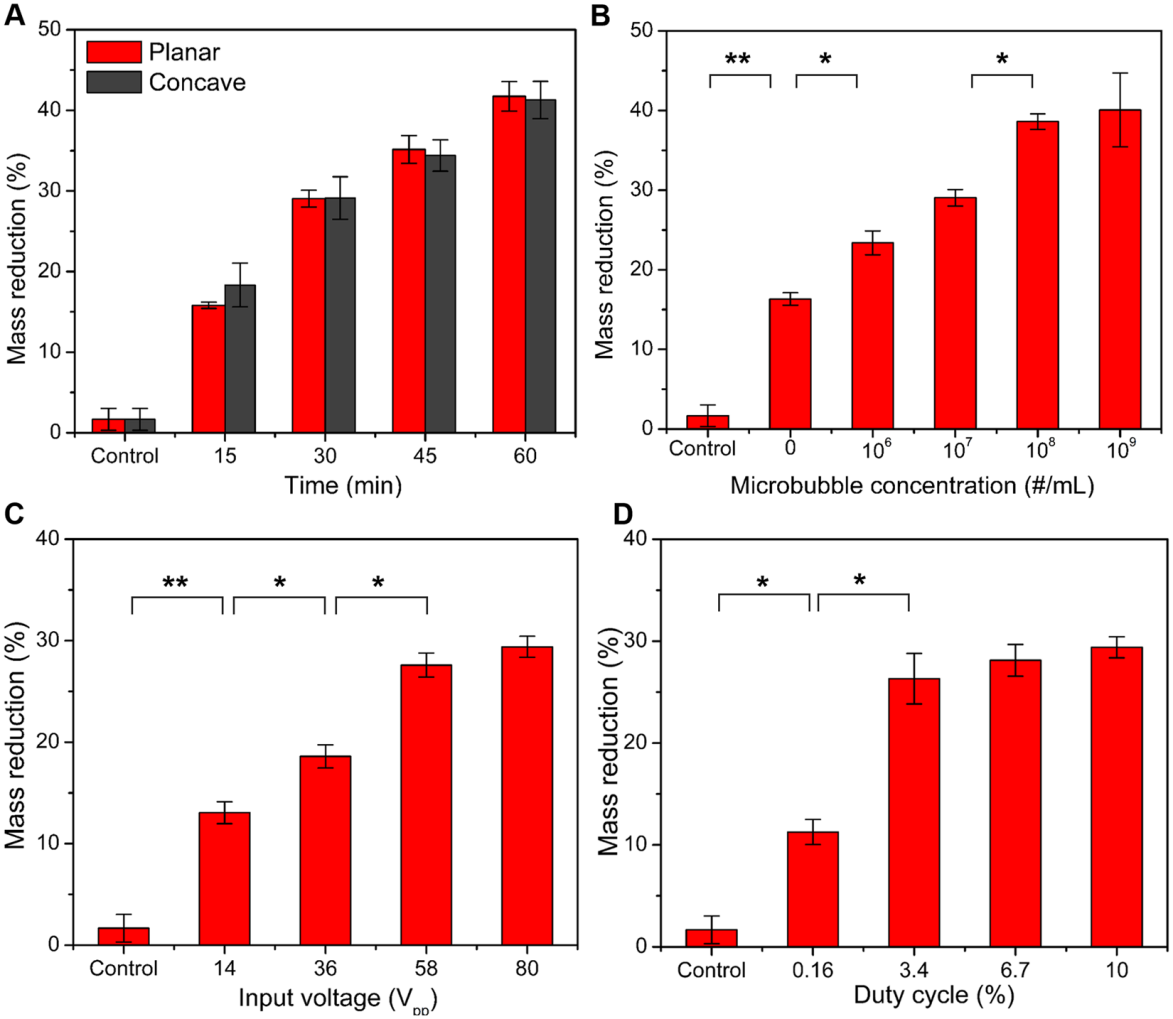
*In vitro* test results with different input parameter. (**A**) Mass reduction of the clots (200 mg ± 10%) in accordance with treatment time of 15–60 min (620 kHz operating frequency, 80 V_pp_ input voltage, 10% duty cycle, 100 μl/min injection of 10^7^ bubbles/ml). Despite of the different pressure output (>100% difference) both aperture prototypes realize similar (<5% difference) mass reduction; the difference of mass reduction showed no statistical significance. (**B–D**) Mass reduction upon variation of treatment condition; fixed parameters for each test are 30 min treatment time, 80 V_pp_ input voltage, 10% duty cycle (5 ms burst duration and 305 cycle-burst), 10^7^ bubbles/ml injection with 100 μl/min flow rate (n = 3). (**B**) Variation of microbubble concentration. (**C**) Variation of input voltage. (**D**) Variation of duty cycle. (*P < 0.05, **P < 0.01).

We then conducted further *in vitro* tests with various input parameters. The two different designs of prototype transducers (planar and concave apertures) were used, and the lysis results were compared. With the same input conditions of voltage, duty cycle, microbubble concentration, and microbubble injection rate, the results obtained by the planar transducer and the concave transducer showed an average lytic rate of 0.7%/min and 0.67%/min, respectively. Interestingly, despite the much higher pressure output (>100% higher at the focal point) of the concave transducer, both prototypes realized similar (<5% difference) mass reduction (Fig. 4(A)). Although the confined beam has higher pressure at the cavitation zone, which is determined by spatial volume within the ultrasound beam where the MI is higher than ~0.3, thrombolysis rate of the planar transducer is similar to that of concave transducer, as shown in the Fig. 2(A). Thus, the planar transducer was used for further experiments while varying other parameters. Since we observed from the initial test that intact microbubbles exist when the bubble concentration is too high, we investigated effect of MCA concentration to find the appropriate MCA injection condition (Fig. 4(B)). The percent mass loss increases with the bubble concentration. This is because larger amount of MCA cavitation can generate larger exposure area of microstreaming-induced shear force^29, 30, 42^. We also found that the higher percent mass loss was achieved with the higher voltage inputs and higher duty cycle (Fig. 4(C) and (D)). The *in vitro* test results with various input parameters had a similar tendency with the previously reported sonothrombolysis results obtained by using a commercially available diagnostic 2.5-MHz transducer^24^.

**Figure 5:**
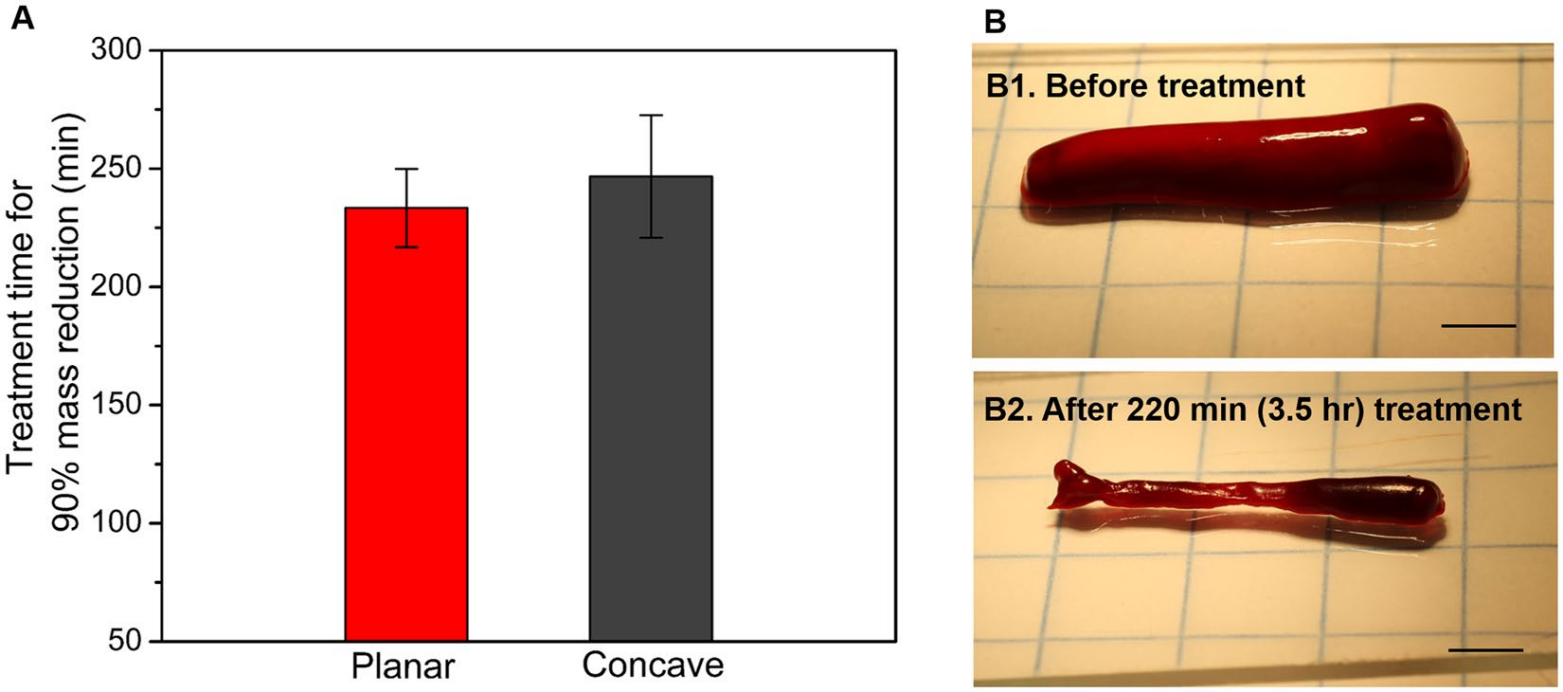
*In vitro* test result for complete mass reduction. (**A**) Required treatment time for 90% mass reduction of 200 mg clot samples with the treatment conditions of 80 V_pp_ input voltage, 10% duty cycle, and 10^7^ bubbles/ml injection with 100 μl/min flow rate (n = 3). The difference of treatment time between planar and concave prototypes showed no statistical significance. (**B**) A 200 mg clot sample decreased in volume and mass after 3.5 hr treatment (scale bar = 3 mm). The remaining small (~20 mg) fibrin (B2) was not completely removed by continued treatment (>4.5 hr).

Next, we investigated the required treatment time to reach 100% mass reduction. The two designs of prototyped transducers were used in these tests. The operating parameters used were determined based on the previous parameter study results presented in Fig. 4(A). First, we observed that the complete thrombolysis was not achieved with only microbubble-mediated sonothrombolysis treatment. After about 3.5 h treatment, red cells inside the clot were almost entirely lysed but some of the fibrin structure remained. Even with a treatment time longer than 4.5 h, the fibrin network was not completely removed (Fig. 5(B)). Hence, further tests were conducted until 90% mass reduction, and the required treatment time was analyzed. As we found in the previous *in vitro* test results, the planar and concave transducers showed similar (approximately 5.2% difference) treatment time for 90% mass reduction. This was expected due to the similar cavitation volume (Fig. 2(A)) of both the planar and concave transducers. Since the fibrin fibers are cleaved by the active enzyme plasmin^43, 44^, we hypothesize that 100% thrombolysis might be expected with a local administration of a minimum amount of thrombolytic agent. Regardless of the improved penetration of rt-PA to the clot, we confirmed that the microbubble-mediated intravascular sonothrombolysis by using our miniaturized transducers can realize a lytic rate of 0.7 ± 0.15%/min.

## Discussion

A catheter-mounted, forward-looking, low-frequency, intravascular transducer was developed and microbubble-mediated clot dissolution was performed *in vitro* using our device in the absence of thrombolytic drugs. We demonstrate that the miniaturized transducers (designed for 8 F catheter) combined with MCA-injection can realize an average lytic rate of 0.7 ± 0.15%/min without the use of rt-PA, unlike the previous microbubble-mediated test result showing only negligible lytic rate (approximately 0.02%/min) by Ekosonic without rt-PA^25^. Due to the reduced cavitation threshold resulting from the presence of MCA, locally delivered acoustic energy by using our device incurs cavitation-induced microstreaming near the clot boundary and realizes red-cell lysis. Note that no ultrasound-induced heating effects were involved in this treatment, only cavitation produced the reported lytic rate without the use of any thrombolytic agent. Conversely, conventional side-looking, intravascular sonothrombolysis transducers for ultrasound-enhanced-fibrinolysis (e.g. Ekosonic) previously produced no decrease in clot mass in the absence of lytic agent (percent mass loss of only 0.95 ± 1.33% by 45 min treatment)^25^. The main features of our device compared to the conventional sonothrombolysis catheter are lower-operating frequency (620 kHz vs. 1.7–2.2 MHz)^17, 25^, higher acoustic intensity (*I*_SPTA_ up to 11 W/cm^2^ vs. 0.5–4.9 W/cm^2^)^25, 45^, and reduced cavitation threshold near the clot boundary due to locally injected microbubbles (<1 MPa vs. ~4 MPa)^25^.

Because these transducers operate at a frequency lower than the resonance frequency range of conventional MCA (1–5 MHz)^39^, the immediate rupture of microbubbles was observed at insonation with relatively low PNP of 200 kPa, corresponding to an MI of 0.27. Although it is unclear whether stable and inertial cavitation have different effects on the improved mass reduction in this study, the higher percent mass loss was achieved with the higher pressure output. The approximate inertial cavitation threshold at this frequency is expected to occur at a PNP of 200 kPa, hence the insonation with the voltage input higher than 40 V_pp_ (corresponding PNP of 200 kPa) yields inertial cavitation of injected microbubbles. In comparison with the treatment case with an input voltage of 80 V_pp_ (corresponding PNP of 350 kPa), the 14 V_pp_ case (corresponding PNP of 100 kPa) exhibited only 45% lytic improvement. Thus, the volume of the cavitation zone within the insonation field is crucial for the lytic rate. The planar and focused aperture transducers yielded similar (<5% difference) average thrombolysis rate and the difference between two different aperture prototypes showed no statistical significance, although the maximum pressure output of the concave transducer is approximately 2 times higher than that of the planar transducer. The reason may be that the concave aperture has a tighter focus that would need to be moved to different regions of the clot in order to generate inertial cavitation of microbubbles. Thus, one further study includes design optimization the custom concave lens with appropriate *f*-number in order to attain the largest cavitation volume with minimized exposure of acoustic energy to the vessel wall.

Considering microbubble behavior within the cavitation zone (Fig. 2(A)), most bubbles are expected to either 1) remain within the cavitation zone where their shells rupture (visibly destroyed microbubbles) and they are replaced by new microbubbles from the tube, or 2) exit this zone intact (visibly undestroyed microbubbles, Fig. 3(A)). Coalescence may also occur in the cavitation zone^46^, however, ultrasound-induced coalescence would be expected to occur rapidly, within the first few microseconds^41^, meaning these coalesced microbubbles would still be in the cavitation zone, where they would be subject to repeated acoustic pulsing and have a high likelihood of destruction^41, 47^. Because the tube delivers microbubbles to the face of the transducer, the microbubble concentration in this zone will be approximately equal to the concentration within the tube.

Furthermore, the transmitted acoustic energy is sufficient to achieve lysis by ultrasound alone, and micro-bubble mediated treatment yielded a lytic rate at least 2 times higher. Based on these experiments, concentrations higher than 10^8^/ml did not produce additional improvement in mass reduction. The chosen concentration of microbubbles in this work is 10^7^ particles/ml, which is in the range of concentrations previously used by others (10^4^ to 6 × 10^8^ particles/ml)^16, 26^. One advantage of our device is that microbubbles are infused locally at the site of the thrombus via a catheter, which means that the injected dose is similar to or less than the bolus dose injected for human imaging (e.g. ~7 × 10^9^ microbubbles/ml for a 70-kg patient, Definity, Lantheus Medical Imaging, North Billerica, Massachusetts, formerly Bristol-Myers Squibb Medical Imaging). With a bubble concentration of 10^7^/ml, the average thrombolysis rate of our system is 1.4 ± 0.33 mg/min. However, the demonstrated thrombolysis rate is not the ultimate limit, since this sonothrombolysis system is designed to minimize the use of thrombolytic drugs to avoid distal embolism caused by the fragmented clot particles. This result is promising since recent work on sonothrombolysis has demonstrated that a lytic rate approximately 10 times higher was achieved when a low dose of rt-PA (0.32 μg/ml) was used in conjunction with microbubble-mediated transcutaneous sonothrombolysis^16^. Thus, we hypothesize that faster (>10 times) lysis can be achieved when rt-PA is injected with the microbubbles by using our device.

The limitations of the present study are the absence of a flow system that more accurately mimics physiological blood flow, evaluation of safety of the acoustic output for the vessel wall, and testing with rt-PA. Previously, researchers have demonstrated that the thrombolysis rate of microbubble-mediated sonothrombolysis is also affected by the blood flow rate^16^. Additional shear forces caused by the high flow rate in the vessel increases clot lysis. Although we did not consider these effects of blood flow in this work, we hypothesize that we can achieve higher thrombolysis rate with a flow system that more closely mimics physiological conditions. The motivation behind this work in modifying the design of the transducer from side-looking to forward-looking is related to safety issues for the vessel wall. We have not evaluated the safety of our device for the vessel wall using histology. The possible mechanisms of vessel wall damage (hemorrhage or endothelial cell injury or dysfunction) resulting from use of the presented device may arise due to 1) ultrasound-induced heating (tissue-ablation) and 2) cavitation-induced microstreaming and microjets^46^. First, no increase in temperature caused by ultrasound-induced heating was observed at the treatment location (i.e. acoustic focus) during our *in vitro* study. Second, despite potential safety issues due to using MCA, previous studies have demonstrated that microbubble-mediated therapy (including sonothrombolysis) using MI < 1.9 is safe from hemorrhage^33^ or inflammatory cell infiltration^33, 38^. Third, the direct exposure to ultrasound energy is significantly reduced laterally by our custom lens (e.g. MI < 0.1 was measured in the lateral direction for distances >3 mm from the center of the transducer), which results in negligible acoustic power on the wall lateral to the transducer and thus extremely low risk of incidental ultrasound-induced bioeffects in the vessel wall. However, in the setting of tortuous vessel segment *in vivo*, theoretically, the forward-looking ultrasound energy could potentially point to vessel wall. Thus, further *ex vivo* experiments can be conducted to determine whether the vessel wall is damaged or not. It is promising that 90% lysis was achieved without any thrombolytic drugs, but in order to avoid the possible distal embolism which can be caused by cleaved clot particles, we will continue *in vitro* experiments using rt-PA.

In summary, the sub-megahertz, forward-looking transducers were developed for the microbubble-mediated intravascular sonothrombolysis. The developed custom transducers exhibited a mean thrombolysis rate of 1.4 ± 0.33 mg/min in the absence of a thrombolytic agent. We demonstrated that this type of the transducer can realize mechanical fragmentation of the clots by cavitation-induced microstreaming. We expect that this transducer is advantageous for ultrasound-enhanced fibrinolysis by local infusion of low-dose thrombolytic drugs, hence the ultimate sonothrombolysis treatment can be achieved while minimizing the use of drugs and decreasing treatment times.

## Methods

### Design of the prototype transducers

The stacked-type, intravascular transducers were designed by using commercial finite element analysis (FEA) software (ANSYS Mechanical APDL®, ANSYS® Academic Research, Release 15.0.7, ANSYS, Inc., Canonsburg, PA, USA), since it has been shown that the electromechanical properties of stacked-type piezoelectric resonator can be accurately analyzed using the finite element method^48^. In order to obtain low-electrical impedance for electrical impedance matching and higher output strain of the piezoelectric resonator, a 6-layer stacked resonator was designed to have sub-megahertz resonance frequency. Each layer thickness is 230 μm, and the total thickness is 1.5 mm. Practical use of the concave lens for the focused beam was also analyzed by FEA. Detailed design procedure for the prototype transducers is described in Supplementary material, S1.

### Fabrication and integration of the prototype transducers

230 μm thick PZT-5A plates (Model 200, TRS Technologies, Inc., State College, PA, USA) were stacked and diced to be properly shaped for 8 F catheter mounting. A conductive bond (E-solder 3022) was used as a bonding layer. The bonding layer thickness was 30 μm. Since the multi-layer resonator design has been widely used for the high-amplitude actuator applications, we followed the previously published fabrication procedure^49^. The detailed fabrication procedure is illustrated in Supplementary material, S2. In order to confine the ultrasound beam, a concave lens was fabricated. Aluminum oxide (Al_2_O_3_)/epoxy mixture material was used as a custom lens material. The detailed procedure to fabricate the custom concave lens is described in Supplementary material, S2.

### Preparation of blood clot samples

Bovine blood clot samples were prepared in a similar protocol as described in previous work^50^. Bovine blood was obtained from Densco Marketing, Inc. (Woodstock, IL, USA). The blood was added into the 2.75% W/V calcium chloride (CaCl_2_) solution (Fisher Scientific Fair Lawn NJ) as coagulant for clotting with a volume ratio of 10:1 (5 ml/50 ml blood). The blood was mixed and transferred to tygon tubes (6.35 mm ID, 7.94 mm OD). The blood-filled tubes were immersed in a 37 °C water bath for 3 h. After clot formation, the tubes were stored at 4 °C for over 72 h for complete retraction^16^. For *in vitro* tests, each clot sample was prepared to have a mass of 200 ± 10% mg in a cylindrical shape with a diameter of ~3.5 mm.

### Preparation of ultrasound contrast agents

Microbubble contrast agents were synthesized in-house as previously described^51^. Briefly, lipid solutions were formulated with a 9:1 molar ratio DSPC and DSPE-PEG2000 (Avanti Polar Lipids, Alabaster, AL, USA) in a solution containing propylene glycol 15% (v/v), glycerol 5% (v/v) and phosphate-buffered saline (PBS) 80% (v/v). Next, 1.5-ml aliquots of lipid solution were placed in sealed 3-ml glass vials and the air headspace was exchanged with decafluorobutane gas (Fluoromed, Round Rock, TX, USA). Agitation with a Vialmix device (Lantheus Medical Imaging, N. Billerica, MA, USA) causes microbubbles with decafluorobutane gas cores and phospholipid shells to form spontaneously. Microbubble concentration and diameter were measured via single particle optical techniques (Accusizer 780, Particle Sizing Systems, Santa Barbara, CA, USA). Mean microbubble diameter was 1.1 μm, with an initial concentration (before dilution) of 10^10^ microbubbles/ml^52^.

### Acoustic characterization of the transducers

For the pressure output and beam profile tests, the transducers were positioned in the water tank filled with degassed water. The transducers were driven by 10-cycle sinusoidal inputs at 620 kHz using an arbitrary function generator (AFG3101, Tektronix Inc., Beaverton, OR, USA) connected with a 60 dB radio-frequency amplifier (Model 3200 L, Electronic Navigation Industries Inc., Rochester, NY). A needle hydrophone (HNA-0400, Onda Corp., Sunnyvale, CA, USA) was used to measure a pressure output (as a function of voltage inputs) at 1 mm away from the aperture. During the separate pressure mapping procedure, the hydrophone position was controlled laterally (3 mm) and axially (4.5 mm) using a computer-controlled motion stage (Newport XPS, Irvine, CA, USA) to measure pressure profiles in a 2D plane including radiation axis.

### *In vitro* tests

For each test, a 200 mg ± 10% clot sample was immersed in 500 μl phosphate buffered saline (PBS) solution stored in a tube (Tygon®, ID: 4 mm, OD: 5.6 mm). The tube was submerged in a water tank filled with degassed water (37 °C). The outlet of the tube was positioned out of the water, thus the intravascular transducers were inserted through the opening in the air. The transducers position was controlled by 3-axis motion stage to maintain constant distance between the transducer and the target clot (~0.5 mm). Since the maximum pressure was obtained at the closest measurement limit (0.5 mm) for both planar and concave prototype transducers and there is no significant pressure difference from 0.5 mm to 1 mm (<3.4%, Fig. 2(A)), we kept the transducer less than 0.5 mm from the clot surface for *in vitro* tests to ensure the most efficient insonation volume was maintained within the target clot. For our case with the concave lens, which yields confined beams without a clearly defined focal zone, positioning the transducer distance less than 0.5 mm for both planar and concave prototypes can be considered as a fair condition for comparing thrombolysis performance. Each treatment batch was conducted with a 30 sec break for every 5 min sonication. In order to evaluate the lytic rate (percent mass loss/min) both planar and concave transducers were used. The input parameters for lytic rate evaluation are 620 kHz excitation frequency, 80 V_pp_ input voltage, 10% duty cycle, and 100 μl/min injection of 10^7^ bubbles/ml. During the variation of input parameters, fixed parameters for each test are 30 min treatment time, 80 V_pp_ input voltage, 10% duty cycle (5 ms burst duration and 305 cycle-burst), 10^7^ bubbles/ml injection with 100 μl/min flow rate. All results presented were averaged and expressed as the mean ± SD (n = 3). Statistical analysis was conducted using the MATLAB Statistical Toolbox (Mathworks, Natick, MA, USA). Student’s t-test (one-tailed distribution) or one-way unbalanced ANOVA was utilized to determine statistical significance of tests with different treatment conditions. A *P-*value < 0.05 was considered as a requirement of statistical significance. For the bubble concentration tests, the control group was treated with a pure PBS injection with the same flow rate without any insonation and bubble injection. For other input parameter tests, the control group was treated with the same process only without insonation. For all figures, photographs were taken using a digital SLR camera (Canon EOS Rebel T3) with 50 mm lens (Canon, f/2.5 Compact Macro Lens), cropped and aligned using Microsoft PowerPoint and Adobe Illustrator CC.

## Electronic supplementary material

Supplementary material
