# Supplementary material for: Intravascular forward-looking ultrasound transducers for microbubble-mediated sonothrombolysis
Source: Sci Rep. 2017 Jun 14;7:3454. doi: 10.1038/s41598-017-03492-4 (PMC5471247; doi:10.1038/s41598-017-03492-4)
Supplement: Supplementary file 1 — Supplementary material [file 41598_2017_3492_MOESM1_ESM.pdf]

Intravascular forward-looking ultrasound transducers for microbubble-mediated sonothrombolysis

Jinwook Kim, Brooks D. Lindsey, Wei-Yi Chang, Xuming Dai, Joseph M. Stavas, Paul A. Dayton & Xiaoning Jiang

Supplementary Material

S1. Design of the prototype transducers.

The stacked-type, miniaturized transducers were designed by using commercial finite element analysis (FEA) software (ANSYS Mechanical APDL®, ANSYS® Academic Research, Release 15.0.7, ANSYS, Inc., Canonsburg, PA, USA). The designs with both planar and concave aperture were simulated by using 2-D axis symmetry finite element models (Fig. S1). PZT-5A ceramic plates (230 μm thick) were stacked to form a 6 layer-bar resonator. The lateral dimension and the total thickness are 1.2 mm and 1.5 mm, respectively. The PZT plates were stacked in alternative direction of poling. Material properties of each transducer components are tabulated in Table S1. 2-D harmonic analysis was conducted to estimate the beam diameter at the designed operating frequency (~600 kHz). The infinite boundary condition (no impedance boundary) was applied at the outer edge of the water medium, hence there was no wave reflection at the water medium boundary. The simulated results showed that the concave lens can confine the beam despite of the small aperture area in terms of the wavelength. The simulated -6 dB beam width of the concave aperture was 51% of the planar aperture beam width. For further design optimization of the transducers in the aspect of the operating frequency and the insonation diameters, the aperture size and the radius-of-curvature can be adjusted.

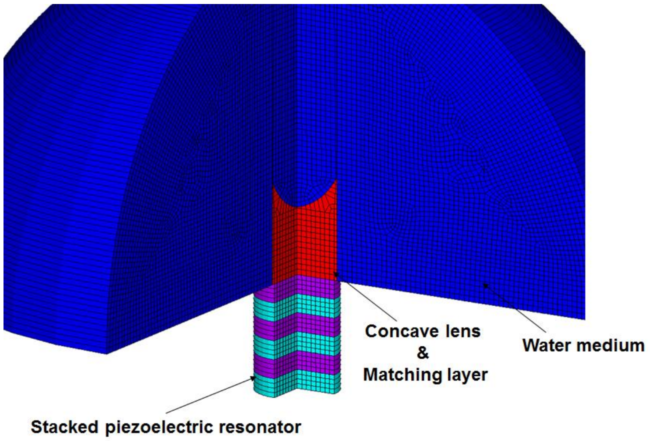

Figure S1. Design of the prototype forward-looking transducers: 2-D axis symmetry finite element model. The meshed model was captured using a software option of 'write metafile (invert white/black)' in ANSYS Mechanical APDL®, ANSYS® Academic Research, Release 15.0.7.

Table S1. Material properties of the stacked-type transducer.

	Density (kg/m^3)	c_{33}^E (GPa)	e_{33} (C/m^2)	$\varepsilon_{33}/\varepsilon_0$
PZT-5A	7750	111	15.8	830
	Density (kg/m^3)	Young's modulus (GPa)	Poisson's ratio	
Matching layer & lens	2000	11.5	0.32	

S2. Fabrication of the prototype transducers.

The transducer fabrication procedure is illustrated in Fig. S2. (A). Large size (7 mm x 7 mm) PZT-5A plates were stacked considering the alternative poling direction of each adjacent plate. E-solder was used as a bonding material and electrically conductive material. After the stacked plates were pressed by a custom bonding fixture, the thickness of the cured E-solder bonding layer between two PZT are approximately 50 um. This bonding layer was used as the exposed electrode area during patterning the electrode. A large size stacked bar was diced to the designed lateral dimension (1.2 mm), and then the insulation layer was attached at the side of each bar resonator. A thin parylene-C layer (20 µm thick) was attached to cover the exposed electrode area. After that, the alternative electrode layers were connected. The electrode-patterned prototype transducer is shown in Fig. S2. (B).

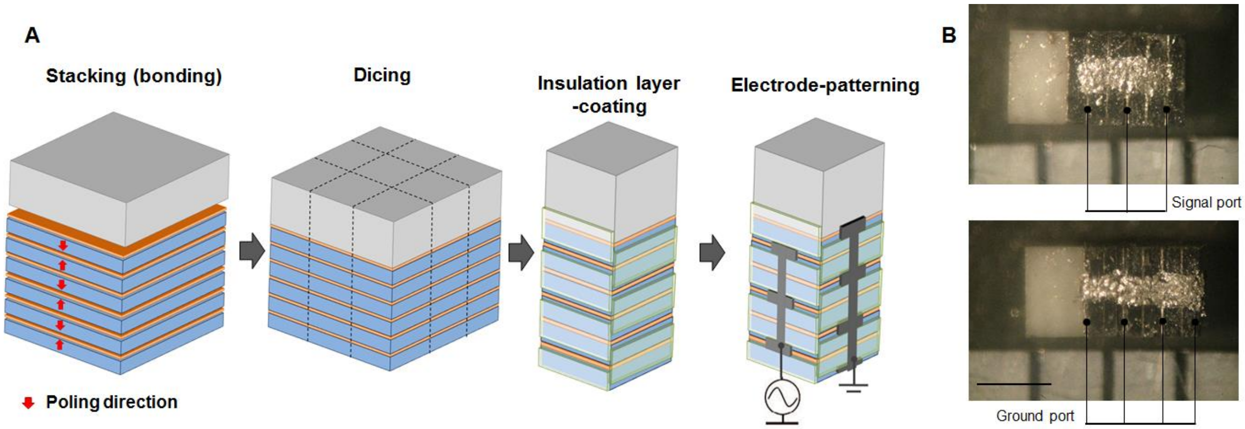

Figure S2. Fabrication of the prototype transducers. (A) fabrication procedure. The figure is drawn by J.K. using Microsoft PowerPoint. (B) custom prototype transducer after electrode-patterning (scale bar = 1 mm). The photographs were captured using a digital SLR camera (Canon EOS Rebel T3) with 50 mm lens (Canon, f/2.5 Compact Macro Lens), cropped and aligned using Microsoft PowerPoint.

The custom concave lens was fabricated following the procedure depicted in Fig. S3. Firstly, a concave-shape polydimethylsiloxane (PDMS) mold was prepared using a steel ball (2 mm diameter). A cured PDMS mold was used to make a PDMS hemisphere. In this process, the mixing volume ratio of curing agent and base was changed from 1:10 to 4:10 for easier separation of the hemisphere from the mold. The prepared hemisphere was used as a shaping tool of a concave lens made of Al_2O_3/epoxy having a radius-of-curvature of 1 mm. *f*-number can be controlled by using different size of a steel ball. The fabricated lens was attached to the transducer using uncured Al_2O_3/epoxy bond.

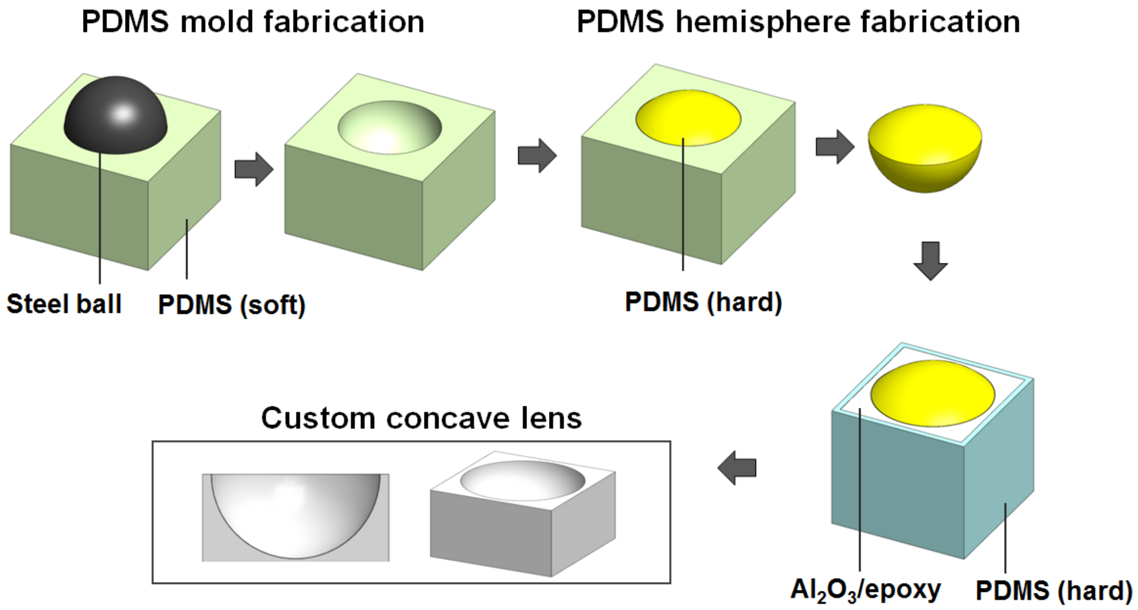

Figure S3. Fabrication of the custom lens. The difference between soft and hard PDMS molds is the mixing volume ratio of curing agent and base. The ratios of soft and hard ones are 1:10 and 4:10, respectively. The figure is drawn by J.K. and W.C. through the combined use of Solidworks Education Edition, Microsoft PowerPoint, and Adobe Illustrator CC.

S3. Cavitation detection

In order to analyze enhanced cavitation effects by injecting MCA near the target clot, microbubble response was measured. With the setup shown in Fig. 1 (C), fundamental excitation signals and microbubble signals were measured by the hydrophone positioned about 5 mm away from the insonation region. The case without MCA injection was also measured to compare it with the case of MCA injection (Fig. S4 (A)). Without microbubble injection, amplitude spectra with various input voltage showed similar level at the harmonic frequency range (1.2-6 MHz). Conversely, measured signals from microbubbles exhibited clear difference with different voltage inputs (Fig. S4 (B)). In the range of 14-58 V_{pp}, discernable nonlinear harmonic signals (2nd to 8th harmonics) were detected whereas significantly reduced (about 20 dB) harmonic components were shown with the 80 V_{pp} input voltage. This result indicates that the inertial cavitation of

all the injected bubbles occurred with the 80 V_{pp} input voltage whereas stable cavitation is dominant with the input voltage lower than 60 V_{pp}.

Table S2. Corresponding PNP and MI values with various voltage inputs.

Volt (V_{pp})	PNP (MPa)	MI
14	0.08	0.10
36	0.20	0.25
58	0.27	0.35
80	0.35	0.45

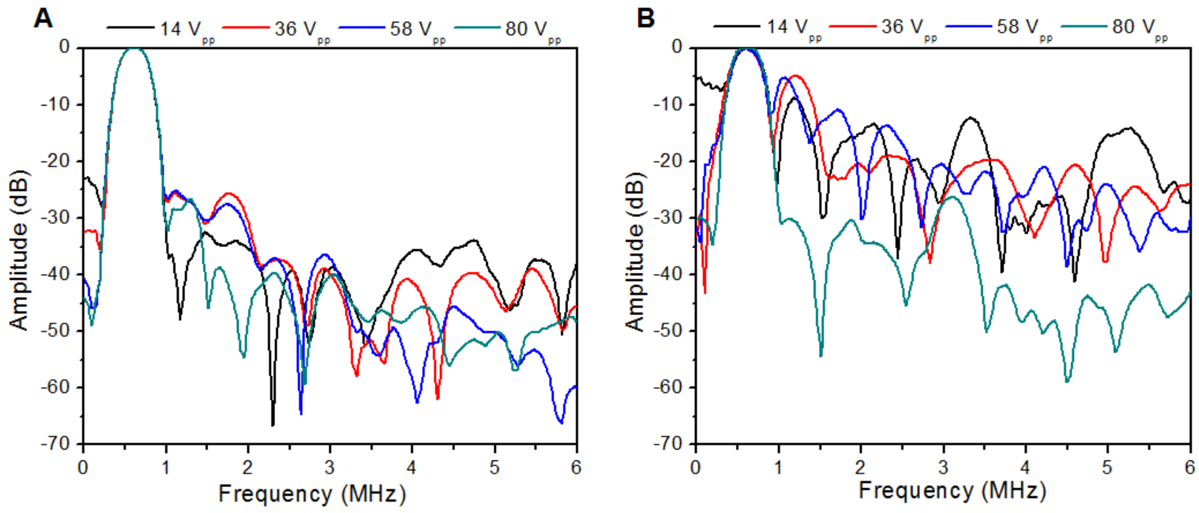

Figure S4. Cavitation detection results. (A) Measured frequency spectra during the treatment without MCA injection. (B) Measured spectra when microbubbles (10^7 bubbles/ml) were injected with 100 µL/min flow rate.

S4. Clot debris

Although our device will be ultimately used for rt-PA combined therapy which is safe from distal embolism, we have investigated particle sizes after the treatment by using a 100 µm filter (sterile cell strainer, 100 µm mesh size, Fisher Scientific, Pittsburgh, PA, USA, Fig. i (left)), since the debris no greater than 100 µm were unlikely to cause hazardous emboli. We have conducted tests with the highest intensity condition among the in vitro test conditions using the concave prototype transducer (620 kHz, 80 V_{pp}, 10% duty cycle with 100 µl/min injection of 10^8 bubbles/ml, 15 min, n=4). After each treatment, the mixture of saline water and melted blood was drained through the filter. The upside of the filter was observed under the microscope (x100). No particle larger than 100 µm was observed for all the test groups. It was not available to evaluate

the detailed percentage of debris particles by this simple test using a one-size mesh. This test result does show that the treatment based on the cavitation-induced microstreaming using our transducer yielded clot fragmentation particles with the size < 100 um.

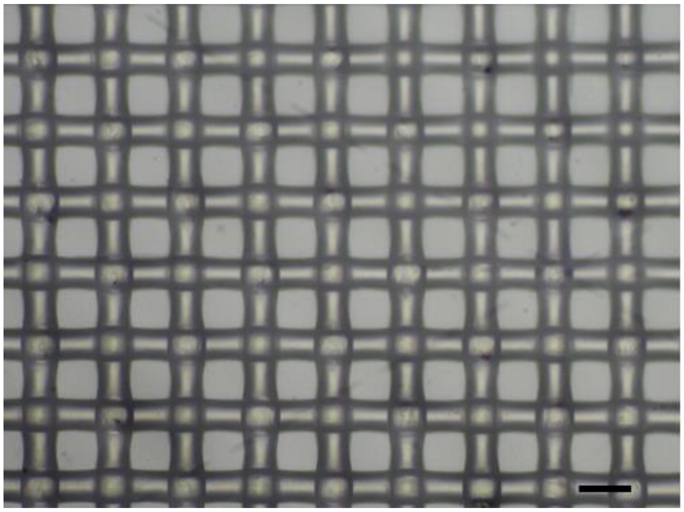

Figure S5. Magnified images of the 100 μm mesh filter (scale bar = 100 μm). The photograph was captured using a digital SLR camera (Canon EOS Rebel T3) with 50 mm lens (Canon, f/2.5 Compact Macro Lens), cropped and aligned using Microsoft PowerPoint.
